# Correlation of plasma N-terminal pro-brain natriuretic peptide (NT-proBNP) with radiographic features of congestion in chest CT scan of patients with COVID-19

**DOI:** 10.1186/s43044-023-00390-1

**Published:** 2023-07-13

**Authors:** Naghmeh Ziaie, Seyed Mohammad Reza Tabatabaie, Khadijeh Ezoji, Ali Bijani, Simin Mouodi

**Affiliations:** 1 Department of Cardiology, Babol University of Medical Sciences, Babol, Iran; 2 Department of Radiology, Babol University of Medical Sciences, Babol, Iran; 3 Social Determinants of Health Research Center, Health Research Institute, Babol University of Medical Sciences, Babol, Iran

**Keywords:** Coronavirus infections, Congestion, Biomarkers, NT-proBNP

## Abstract

**Background:**

Given the importance of chest computed tomography (CT) to differentiate congestion from COVID-19 pneumonia, and considering the association of chest CT findings with cardiac biomarkers in patients with concomitant COVID-19 and heart failure, this study was conducted to identify the correlation between plasma NT-proBNP level and radiographic features of congestion in patients with COVID-19. This retrospective cohort research was carried out on adult hospitalized patients with COVID-19 and the plasma concentration of NT-proBNP was measured. The most important findings in chest CT have been considered to differentiate COVID-19 pneumonia from congestion. The study population was divided into two groups based on the presence of these imaging characteristics.

**Results:**

Totally, 180 patients with a mean age of 59.6 ± 14.6 years were included in the research. The radiographic findings related to congestion have been found in chest CT of 107 (59.4%) patients. Mean plasma concentration of NT-proBNP in patients with and without radiographic features of congestion was 9886.5 ± 12,676 and 2079.9 ± 4209.3 pg/mL, respectively (*p* < 0.001). The area under the curve of plasma levels of NT-proBNP for identification of patients with COVID-19 who had pulmonary vein enlargement in chest CT was 0.765 (95% CI 0.688–0.842) and 0.731 (95% CI 0.648–0.813) for the individuals who had interlobar fissure thickening (*p* < 0.001).

**Conclusions:**

The diagnostic accuracy of plasma NT-proBNP and its positive correlation with radiographic features of congestion in chest CT scan of patients with COVID-19 can be helpful for administering appropriate medications to prevent blood volume overload.

## Background

Various clinical aspects of the disease have been demonstrated in published scientific evidence since the beginning of the novel coronavirus disease (COVID-19) pandemic. The cardiovascular system is one of the target systems involved in patients with the disease [1–3]. The binding of the virus to the membrane-bound form of angiotensin-converting enzyme 2 (ACE2) has been represented as one of the most important mechanisms for the involvement of the cardiovascular system in this disease, as heart is one of the tissues that express ACE2 [4, 5]. Cardiac injury in COVID-19 is diverse, ranging from microangiopathy to myocarditis, myocardial infarction or even heart failure [6]. It has been reported that 12–20% of patients with COVID-19 might have acute myocardial injury, and the patients who develop myocardial injury are expected to have worse outcomes [7, 8]. Limited data are available regarding the occurrence of heart failure in patients with COVID-19; however, a cohort study on 3080 individuals with confirmed COVID-19 who were followed for ≥ 30 days revealed an incidence of acute heart failure as 2.5% in these patients [9].

Pulmonary congestion has been represented as one of the hallmarks of heart failure (HF) [10]. Due to different patterns of pulmonary involvement in COVID-19, early and precise diagnosis of congestion in these patients is challenging [11]. Some literature recommended that chest computed tomography (CT) might provide an integrated heart and lung imaging assessment and differentiate cardiac involvement from COVID-19 pneumonia [12, 13]. N-terminal pro-brain natriuretic peptide (NT-proBNP) is a cardiac biomarker released as a response to hypoxia and ischemia, activation of the sympathetic system, systemic inflammation, myocardial stress and increased ventricular wall tension [14, 15]. It has been reported as a marker of reduced left ventricular systolic function, severe condition and poor prognosis in patients with heart failure [8]. Although elevated NT-proBNP level has been presented as an independent factor associated with an increased mortality rate in patients with COVID-19 [8, 16], there is no definite cut-off point for NT-proBNP level that can properly indicate congestion in COVID-19 patients.

A few studies have examined the association of chest CT findings with cardiac biomarkers in patients with concomitant COVID-19 and congestion [17]. Because fluid administration should be done more carefully in patients with congestion, it is necessary to identify this group of patients as soon as possible to have more precise control of fluid input and output. On the other hand, diagnostic procedures in which the physician's exposure time with patients with COVID-19 is high are less recommended, thus increasing the value of laboratory diagnostic methods. This research was conducted to assess the correlation between plasma NT-proBNP levels and radiographic features of congestion in chest CT scan of patients with COVID‐19.

## Methods

### Study design, setting and participants

This observational retrospective cohort research was performed on adult hospitalized patients with COVID-19 diagnosis. Inclusion criteria were: (1) adult patients whose COVID-19 infection was confirmed with real-time polymerase chain reaction assay, or combination of clinical manifestations and chest CT findings, (2) the plasma levels of NT-proBNP were measured, and (3) have been admitted in the state hospitals affiliated to Babol University of Medical Sciences, Babol, Iran, from February 20, 2020, to July 21, 2020. A convenience sampling was conducted, and the patients whose plasma levels of NT-proBNP were not measured were not included in the study. All patients or their proxies provided a written informed consent form to participate in this research. The study protocol was approved by the Ethics Committee of Babol University of Medical Sciences, Iran, with the registration code: IR.MUBABOL.REC.1399.164.

### Outcomes and variable assessment

Demographic characteristics, including age and gender, previous history of cardiovascular disorders, any other comorbidities, laboratory findings and final outcome (discharge from the hospital or death), were recorded in the research datasheet. Chest CT scans were examined by two blind radiologists separately. A previous study recommended five important findings in chest CT to differentiate COVID-19 pneumonia from congestion. They include (1) cardiac enlargement, (2) peribronchovascular thickening, (3) thickening of the interlobular fissure, (4) upper pulmonary vein enlargement and (5) subpleural effusion [12]. The study population was divided into two groups: the case group (with radiographic features of congestion) included the patients who had these findings in chest CT, and another group was the patients without these imaging characteristics [18]. The plasma levels of NT-proBNP were measured by ELISA in a special laboratory with the RAMP device.

### Statistical methods

Data analysis was performed using SPSS-16 software package. Categorical data such as distribution of different chest CT findings were described using frequencies and percentages, and quantitative data, including plasma levels of NT-proBNP, were presented with mean and standard deviation. The plasma concentration of NT-proBNP that did not have a normal distribution was analyzed using the Mann–Whitney *U* test. Chi-square, *t* test and Pearson's correlation test were used for data analysis. Receiver operator characteristic (ROC) curve and area under the curve were used to describe the sensitivity and specificity of NT-proBNP for identification of congestion and to examine the association of NT-proBNP and radiographic features of congestion in patients with COVID-19. P-value of less than 0.05 was considered statistically significant.

## Results

Totally, 180 patients (96; 53.3% male and 84; 46.7% female) with COVID-19 diagnosis were included in the research. Mean age of the participants was 59.6 ± 14.6 (a range of age from 20 to 90) years and did not have any significant difference between men and women (*p* = 0.131). The number and percent of different lesions in chest CT scan were as follows: subpleural effusion (173 individuals; 96.1%), ground-glass opacities (163; 90.6%), cardiomegaly (127; 70.6%), peribronchovascular thickening (113; 62.8%), pulmonary consolidation (85; 47.2%), interlobular septal thickening (84; 46.7%), thickening of the pleura (62; 34.4%), pulmonary vein enlargement (58; 32.2%), crazy paving pattern (53; 29.4%), interlobar fissure thickening (48; 26.7%), pericardial effusion (41; 22.8%), air bronchogram (39; 21.7%), lymphadenopathy (10; 5.6%), and bronchiectasis (9; 5.0%). Pulmonary involvement in 37.2% of patients had a peripheral pattern, 5.0% had a central pattern, and 46.1% had both of them. Subpleural effusion showed a bilateral pattern in most (80.6%) patients.

Out of them, the radiographic findings related to heart failure have been found in chest CT of 107 (59.4%) patients; and 73 (40.6%) individuals did not have this evidence. The baseline characteristics of these patients are presented in Table 1. Fifty-five (51.4%) of the patients who had radiographic evidence of congestion were female, and 52 individuals (48.6%) were male (*p* = 0.131); 25.0% of them reported a previous history of congestive heart failure (*p* = 0.120); 28.6% and 23.4% had a previous history of diabetes mellitus (*p* = 1.0) and coronary artery disease (*p* = 0.303), respectively. Mean left ventricular ejection fraction (LVEF) in patients with and without radiographic features of congestion in chest CT was 46.1% ± 10.8% and 51.5% ± 7.2%, respectively (*p* < 0.001). Twenty-two (20.6%) of the patients with radiographic evidence of congestion had LVEF ≤ 35% in echocardiography examination (*p* = 0.005). Systolic pulmonary artery pressure in patients with and without radiographic evidence of congestion was 34.5 ± 13.9 and 27.1 ± 3.9 mmHg, respectively (*p* = 0.182).

**Table 1:** Baseline characteristics of the patients with COVID-19 with/without radiographic features of heart failure in chest CT

Characteristics	With radiographic evidence of heart failure in chest CT *N* = 107	Without radiographic evidence of heart failure in chest CT *N* = 73	*p* value (*t* test)
	Mean ± standard deviation		
Age (year)	61.5 ± 15.5	56.7 ± 12.7	0.030
The initial white blood cell concentration in peripheral venous sample (per microliter)	10,952.3 ± 5703.5	10,176.3 ± 5110.6	0.492
Serum level of ESR (mm/h)	49.3 ± 37.8	58.8 ± 32.9	0.239
Serum CRP (mg/L)	95.2 ± 81.9	97.9 ± 66.0	0.871
Serum creatinine (IU/L)	1.7 ± 1.6	1.5 ± 1.1	0.417
Serum BUN (mg/dL)	35.0 ± 20.6	33.8 ± 34.2	0.826
SpO2 on the first day of hospitalization (%)	90.5 ± 4.8	91.6 ± 5.6	0.161
NT- proBNP (pg/mL)	9886.5 ± 12,676	2079.9 ± 4209.3	< 0.001*

ESR, erythrocyte sedimentation rate; BUN, blood urea nitrogen; CRP, C-reactive protein; SpO2, peripheral capillary oxygen saturation

*Mann–Whitney test was used for data analysis

**Fig. 1:**
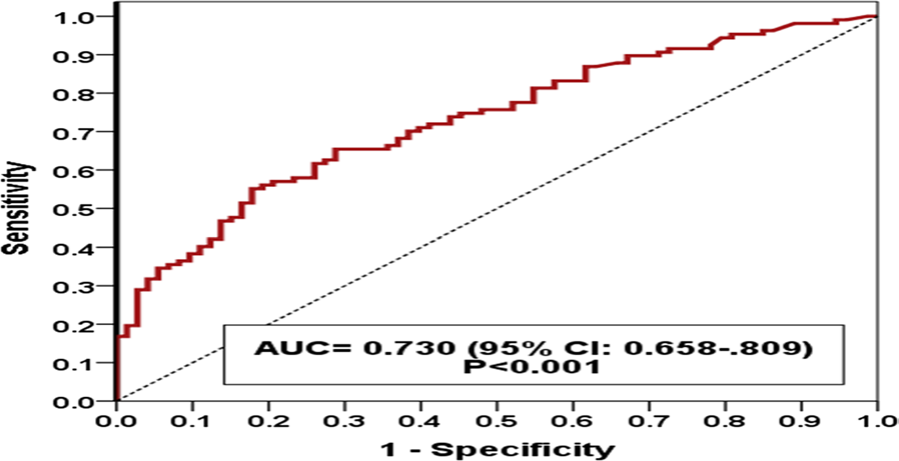
Sensitivity and specificity of NT-proBNP for identification of patients with COVID-19 and radiographic features of heart failure in chest CT

Mean plasma level of NT-proBNP in patients who had increased plasma level of t-troponin was 17,530.2 ± 14,103.4 and in patients with normal range of t-troponin was 3853.3 ± 6813.2 pg/mL (*p* < 0.001). The diagnostic accuracy and area under the ROC curve of plasma levels of NT-proBNP for identification of patients with COVID-19 and five differentiating HF features in chest CT are presented in Table 2 and Fig. 1. The diagnostic accuracy of different plasma levels of NT-proBNP for predicting the occurrence of congestive heart failure in patients with COVID-19 is presented in Table 3. Totally, 31 patients (17 persons with radiographic evidence of heart failure and 14 without this evidence) died (*p* = 0.273).

**Table 2:** Area under the curve of plasma level of NT-proBNP for identification of patients with COVID-19 and five differentiating heart failure features in chest CT

Radiographic features in chest CT	Area under the curve (95% CI)	Standard error	*p* value
Pulmonary vein enlargement	0.765 (0.688–0.842)	0.039	< 0.001
Interlobar fissure thickening	0.731 (0.648–0.813)	0.042	< 0.001
Peribronchovascular thickening	0.678 (0.598–0.758)	0.041	< 0.001
Cardiomegaly	0.675 (0.592–0.759)	0.042	< 0.001
Subpleural effusion	0.519 (0.286–0.752)	0.119	0.865
The five mentioned radiographic features	0.730 (0.658–0.809)	0.037	< 0.001

**Table 3:** Sensitivity, specificity, positive and negative predictive value, and likelihood ratio of different plasma level of NT-proBNP for prediction of congestive heart failure in patients with COVID-19

Plasma level of NT-proBNP (pg/mL)	Sensitivity % (95% CI)	Specificity % (95% CI)	Positive predictive value % (95% CI)	Negative predictive value % (95% CI)	Positive likelihood ratio (95% CI)	Negative likelihood ratio (95% CI)
125	90 (84–95)	29 (18–39)	65 (57–73)	66 (49–82)	1.26 (1.07–1.48)	0.36 (0.18–0.70)
300	83 (76–90)	40 (29–51)	67 (59–75)	62 (48–76)	1.38 (1.12–1.69)	0.42 (0.25–0.70)
450	75 (67–83)	55 (43–66)	71 (62–79)	60 (48–71)	1.65 (1.26–2.18)	0.46 (0.31–0.68)
900	66 (57–75)	63 (52–74)	72 (64–81)	56 (45–67)	1.79 (1.29–2.49)	0.53 (0.39–0.73)
1800	61 (51–70)	74 (64–84)	77 (68–86)	56 (46–66)	2.33 (1.54–3.54)	0.53 (0.40–0.70)
4500	47 (37–56)	85 (77–93)	82 (72–92)	52 (43–61)	3.10 (1.73–5.54)	0.63 (0.51–0.77)
9000	33 (24–42)	95 (89–100)	90 (80–99)	49 (41–57)	5.97 (2.22–16.08)	0.71 (0.62–0.82)

## Discussion

Nearly 60% of the examined hospitalized patients with COVID-19 had radiographic features of congestion in chest computed tomography. This high incidence of congestion may be due to delays between the onset of the disease and hospitalization, when more advanced organ damage related to COVID-19 has occurred, or because the study examinations have been conducted for patients with more severe forms of the disease; also, we included the patients whom the plasma levels of NT-proBNP were measured. Zhu et al.’s study in China demonstrated that ground-glass opacity, consolidation, crazy paving pattern, and interlobular septal thickening had no significant difference between heart failure and COVID-19 pneumonia. However, pulmonary vein enlargement, subpleural effusion, cardiomegaly, peribronchovascular thickening, and interlobar fissure thickening have been observed more frequently in heart failure group than that in COVID-19 group [12].

Although there are some similarities in imaging features of congestive heart failure and COVID-19 pneumonia, early cardiologic assessment of the patients with radiographic evidence of heart failure should be considered by the management team of patients with COVID-19, so that appropriate care can be started earlier and serious complications of congestion can be prevented. Furthermore, Heuvel et al. suggested a limited value of standard echocardiography examination for routine screening of cardiac function in hospitalized COVID-19 patients [6], and this recommendation can multiply the importance and necessity of paying attention to radiographic characteristics of congestion in chest CT.

Mean plasma concentration of NT-proBNP was about 10,000 pg/mL in COVID-19 patients with heart failure imaging features. A recent systematic review represented this natriuretic peptide as the most commonly used systemic biomarker to distinguish acute lung injury from cardiogenic pulmonary edema, and the area under the curve for its diagnostic accuracy has been reported from 0.67 to 0.83 [19]. However, a variable predictive ability of NT-proBNP for identifying heart failure has been reported in previous studies. Some of this variability is due to its decrease after treatment for heart failure. Pranata et al. reported a sensitivity of 76% (46%-92%) and specificity of 88% (71%–96%) for an association between NT-proBNP and mortality in patients with COVID-19 [8]. Gao et al. reported a plasma level higher than 88.64 pg/mL to be related to a lower survival rate of patients with COVID-19 [16]. Heuvel et al. examined the plasma levels of NT-proBNP in hospitalized confirmed COVID-19 patients and reported that 8% of the patients had an NT-proBNP value of higher than 1,000 pg/mL, and the maximum NT-proBNP level was reported as 25,000 pg/mL [6]. Hill recommended the age-adjusted cut-off point of 900 pg/mL for plasma levels of NT-proBNP for diagnosis of heart failure in 50–75 years patients [20]. Considering the sensitivity and specificity of NT-proBNP, it should be measured in the early stages of heart involvement before starting treatment of heart failure, to be more helpful in diagnosis [19].

The diagnostic accuracy of plasma levels of NT-proBNP and its positive correlation with radiographic features of congestive heart failure in chest CT scan may be useful in patients who have not decreased levels of EF. Particularly in initial stages of heart failure, patients may experience higher NT-proBNP even with normal EF. Due to our findings about the diagnostic accuracy of different plasma levels of NT-proBNP to predict the congestion, it is recommended to have more precise control of fluid input and output for COVID-19 patients with higher levels of NT-proBNP (especially, those who had plasma levels of NT-proBNP higher than 300 pg/mL), and to administer appropriate medications -such as diuretics- to prevent blood volume overload, if needed. Hospitalized patients with COVID-19 diagnosis may receive different intravenous infusions, and if the fluid input and output are not controlled, a higher incidence of congestive heart failure will be expected to occur. In our study, the patients with imaging findings suggestive of congestion were older than the other group. Similar to our finding, Rey et al. reported higher age in patients with heart failure [9]. Gao et al. reported patients with severe COVID-19 who had higher NT-proBNP levels were older, and higher levels of systematic inflammation biomarkers and increased cardiac injury markers were observed. Older age and to have underlying cardiac disorders predispose COVID-19 patients to have more severe conditions and poorer prognosis [21].

In this research, t-troponin and NT-proBNP showed a significant association; however, other examined proinflammatory markers such as ESR, and C-reactive protein had no significant difference between patients with versus those without radiographic features of congestion. Guo et al. assessed 187 patients with confirmed COVID-19 infection and showed that T-troponin levels had a positive linear correlation with plasma NT-proBNP levels. During hospitalization, patients with elevated NT-proBNP and t-troponin levels are expected to have more serious complications and worse clinical outcomes [7]. We included the patients with COVID-19 who had NT-proBNP assessment. In addition, left ventricular systolic function of the patients prior to COVID-19 diagnosis and also the severity of COVID-19 infection have not been examined. These can be mentioned as the most important limitations of this study.

## Conclusions

The diagnostic accuracy of plasma concentration of NT-proBNP and its positive correlation with radiographic features of congestive heart failure in chest CT scan of patients with COVID‐19 can be helpful at early stages of cardiac involvement before starting the treatment of heart failure.

## Data Availability

The datasets used and/or analyzed during the current study are available from the corresponding author on reasonable request.

## Abbreviations

CT: Computed tomography
COVID-19: Novel coronavirus disease
NT-proBNP: N-terminal pro-brain natriuretic peptide
ROC: Receiver operator characteristic
